# Supplementary material for: Chromothripsis during telomere crisis is independent of NHEJ, and consistent with a replicative origin
Source: Genome Res. 2019 May;29(5):737–49. doi: 10.1101/gr.240705.118 (PMC6499312; doi:10.1101/gr.240705.118)
Supplement: Supplemental Material [file supp_gr.240705.118_Supplemental_file_1.zip › contigs/annotated_contigs/DB76/contig.2.DB76_length_783_mean_cov_8.37164750958.docx]

**DB76_length_783_mean_cov_8.37164750958**

```
GGGTGCGGGTGCGGAGGGCTCGCGCTGCTGGGCACCCATGGACCTCAGCCACGGCGGGCCCAGGGACGGACCTCCAGGAGGCCTGCTGG
 >chr17:73839451-73839840 - E=3e-219
GGGAACAGGTGCGGGGCATCACTGGGGCTGGAGGCCGGGGTGCTGGGGCCCCCATACCCTTGGCCTGGATCAGGCCTCAGAGGAGCCAT

TCCTGTCCATCTGAGCCTGCTCTGGGCCTCCCGGGACACTGCCTTTCCACCTTGCTCTGCAGATCCAGCCTCCATCCCACCACTTCTCC

CCCGAGCAGGTAAGAGTCAGAGTGCTGGGGTGCAGAGAGGTGGGGAGGGCGGCTGTGTGTCCTCCTGCCGGCCGATGCTGATGGCAGCA

AGCAACTTGCGGTGGGGAGGGGACTCCTGGGT|C|AACAACAGAGAATCCCACAAAGAAACCATACACTTCCTATAACCAATGTTCCCC
                                 >chr17:73781804-73782199 - E=9e-223
AACCCCAGAACAGTTTCCCAAGGTCTTCTATAACAAGAGTTCCTCCTGACTGGTTTGAGTTCTTTCTACTCCAACAGATGATTCTGTTC

TTCGTTCACTGAACAACTTAAGAGCTGTTCAACCCCCCAGGTTCCAAAACCTCTCGCAGCTGGGGATCCTTTTAAACATGCACATCACT

CAGGAGCCCATGGGGATGAGAGGCATTCACAGAGCATCAGGGAAAATGGAAAGCAACATTTTGCTTAACAGGAGAAACCGCCCCTTATT

CATCTTTCTCCCACATCACCTAACTCTAGGGAATGCTTAACGTGGCTTGAAGGAAATGAAGACAGCGACCTAA
```
